# Formulation and Evaluation of Thermosensitive Biogels for Nose to Brain Delivery of Doxepin

**DOI:** 10.1155/2014/847547

**Published:** 2014-06-18

**Authors:** Anuja Naik, Hema Nair

**Affiliations:** Department of Pharmaceutics, Bombay College of Pharmacy, Kalina, Santacruz (E), Mumbai 400 098, India

## Abstract

Thermoreversible biogels can serve as effective systems for delivery of drugs through nose with increased nasal residence time. The objective of this study was to use chitosan and glycerophosphate based thermoreversible systems for delivery of doxepin to brain through intranasal administration. Formulations were prepared by admixture of suitable dilutions of chitosan and glycerophosphate with or without polyethylene glycol, followed by addition of the antidepressant doxepin hydrochloride. Both systems were evaluated for gelling characteristics, rheology, mucoadhesion, *in vitro* release, and *ex vivo* permeation through sheep nasal mucosa. *In vivo* efficacy was evaluated in Swiss albino mice through the forced swim test. Nasal tissues of mice subjected to repeated exposure to formulation were evaluated histopathologically. Both formulations gelled rapidly at 37°C, returned to sol state on cooling, and exhibited thixotropy. Addition of polyethylene glycol decreased the glycerophosphate content required for gelation and rendered the formulation isotonic. Both gels showed good mucoadhesion, enhanced drug permeation, and provided prolonged *in vitro* release at 37°C. Efficacy of the formulation in treated groups was inferred from the measured pharmacodynamic parameter and histopathological reports of formulation treated groups showed no significant local toxicity. The biogels could be potential systems for effective drug delivery to brain via nose.

## 1. Introduction

Drug delivery through the nasal route has been used for the treatment of local diseases such as nasal congestion, allergies, and infections and, in addition, also for systemic delivery [1]. The nasal mucosa offers numerous benefits as a target tissue for drug delivery, such as rapid onset of drug action, plasma drug profiles resembling i.v. infusions, potential for central nervous system delivery, and bypass of first-pass metabolism [2, 3]. However, drug delivery via nasal mucosa is also associated with certain drawbacks including drug loss due to rapid mucociliary clearance resulting in a short residence time of the formulation, potential for ciliotoxicity, enzymatic degradation, and relatively smaller surface area of absorption. Attempts have been made to overcome these drawbacks and to increase the nasal bioavailability of drugs through use of bioadhesives, permeation enhancers,* in situ* gelling systems, microspheres, and nanoparticles, among others.* In situ *gelling systems offer a special advantage in that these systems, being liquid at room temperature, offer ease of administration followed by rapid gelling at body temperature resulting in longer duration of residence and possibility of sustained release of drug. Thermoreversibly gelling systems for intranasal delivery of drugs have been reported by several authors. Various polymers including HPMC, poloxamer, carbopol, sodium carboxy methyl cellulose, sodium alginate, and chitosan have been employed alone or in combination and developed for intranasal delivery of actives including ropinirole [4], sumatriptan [5], salbutamol sulphate [6], metoclopramide [7], fexofenadine HCl [8], and diltiazem HCl [9]. The systems have resulted in several benefits including enhanced permeation and absorption across nasal membrane, improved bioavailability, and/or prolonged delivery.

Products designed for nasal administration and intended for systemic delivery of actives have also found commercial acceptance such as butorphanol tartrate spray, cyanocobalamin nasal gel and spray, desmopressin nasal spray, zolmitriptan spray, fluticasone spray, and hydrocortisone nasal drops. In addition, studies have also shown the suitability of the nasal route for preferential targeting of drugs to the central nervous system (CNS) [1] bypassing the blood brain barrier (BBB). For management of CNS disorders, the requirement of the drug to cross the BBB and enter into cranial circulation and into cranial interstitial fluid is a major hindrance. Several approaches such as disruption of BBB, receptor mediated transport, use of cell penetrating peptides, and targeted delivery using prodrugs have been investigated to target drugs to CNS. In recent times, interest has been generated for delivery via the nasal route as a viable alternative for achieving adequate levels in the cranial circulation and this has been investigated for a number of agents, for example, carbamazepine, sumatriptan, metoclopramide, doxylamine, and morphine [10]. Nose to brain pathway involves transport of drugs via olfactory pathway [11]. The olfactory pathway is situated below the cribriform plate of the ethmoid bone which separates the cranial cavity from the nasal cavity. The olfactory neurons penetrate the cribiform plate which is surrounded by arachnoid membrane containing subarachnoid cerebrospinal fluid (CSF) between the nerve and the membrane. This terminates as the olfactory sensory endings, which penetrates through the olfactory mucosa. Therefore the drugs can enter into the brain parenchyma via CSF [12]. Transport can occur through three pathways, that is, transcellularly between the sustentacular cells by endocytosis for lipophilic drugs, paracellularly through tight junctions between the sustentacular cells, and intracellular axonal transport via olfactory nerve pathway following endocytosis or pinocytosis into olfactory bulb.

Chitosan is a GRAS certified natural cationic polymer, soluble in dilute aqueous acids and has a number of unique properties such as antimicrobial activity, biocompatibility, and biodegradability, which attracts scientific and industrial interest in such fields as biotechnology, pharmaceutics, cosmetics, food science, and textiles. The polymer has been employed in the nasal delivery of proteins, peptides, and vaccines in the form of microspheres and nanoparticulates [13–16]. It is especially useful via this route due to several beneficial properties including absorption enhancement via transient opening of tight junctions of nasal epithelial cells and mucoadhesiveness.

pH sensitive chitosan solutions can be transformed to temperature sensitive gelling systems by addition of glycerophosphate [17]. Glycerophosphate (GP) is a weakly basic organic compound naturally found in the body with FDA approved intravenous use as a source of phosphate in the treatment of unbalance of phosphate metabolism. For the present study GP is employed as the disodium salt of beta-glycerophosphate.

Doxepin hydrochloride (*N*,* N*-dimethyldibenz[*b*,*e*]oxepin-Δ*11[*6*H],* *γ*-propylamine hydrochloride) is an agent used in the treatment of both mild and moderate depression. It potentiates the effect of biogenic amines in brain by inhibiting their reuptake. It also inhibits reuptake of norepinephrine. Oral doxepin is extensively metabolized by first-pass elimination, desmethyldoxepin being the major active metabolite. The oral bioavailability of doxepin is poor and variable (13–45%).

The present work details studies on formulation and evaluation of a novel chitosan-GP based thermosensitive gel incorporating doxepin for intranasal administration. The prepared systems were evaluated for different parameters like gelling characteristics, rheological characteristics,* in vitro* drug release, and* ex vivo *permeation across sheep nasal mucosa. The systems were also evaluated* in vivo* in mice for activity of nasally administered doxepin and via histopathology for nasal mucosal irritation.

## 2. Materials and Methods

Chitosan (degree of deacetylation ~89%) was supplied by Central Institute of Fisheries Technology (Cochin, India), *β*-glycerophosphate (GP) was purchased from Central Drug House (Delhi, India), polyethylene glycol (PEG) 4000 was purchased from S.D. Fine Chem. (Mumbai, India), and doxepin hydrochloride (Dox) was kindly provided as gift sample by Torrent Pharma Ltd. (Gujarat, India). Porcine mucin was obtained from Sigma-Aldrich. All other chemicals and reagents used in the study were of analytical grade.

### 2.1. Preparation of Gels

All thermoreversibly gelling systems were prepared by simple admixture of components under suitable conditions. Chitosan solution in 0.1 N HCl (2.5% w/v, 4 mL) and aqueous GP solution were separately prepared and cooled to 4°C. This was followed by dropwise addition of 1 mL of GP into the chitosan solution with vortexing. Two types of gelling systems were prepared: those containing chitosan and GP at higher concentration of GP and those containing chitosan, GP, and, in addition, PEG 4000. The final mixtures contained 10% w/w of GP and 2% w/v chitosan (C-GP) for the former system while the latter contained 4.5% w/v of GP, 2% w/v chitosan, and 1% w/v PEG (C-GP-PEG). Next, Dox (5 mg/mL) was added to the formulations and dissolved completely followed by addition of PEG in case of the PEG containing sols to obtain C-GP-Dox and C-GP-PEG-Dox, respectively.

### 2.2. Characterisation of Gels

*Gelation Characteristics*

#### 2.2.1. Gelation Time, Gelation Temperature, and pH

The gelling temperature of C-GP-Dox and C-GP-PEG-Dox was measured by immersing the sols in a water bath and increasing the temperature gradually from 15°C to 40°C at a rate of 0.5°C/min. The temperature was maintained for 10 min at 15°C, 25°C, 37°C, and 40°C. The tubes were inverted at frequent intervals until movement of the meniscus on tilting of tube was arrested. Gelation time was measured as the time required to stop the flow of gel at 37°C on immersing the solutions in a thermostatic water bath. pH of all the solutions was recorded at room temperature using a standardized pH meter.

#### 2.2.2. Rheological Evaluation

Both C-GP-Dox and C-GP-PEG-Dox formulations were characterized for their rheological properties using Brookfield cone and plate viscometer. The viscosities of the samples were measured for sol and gel states by holding the samples at 25°C and 37°C, respectively. Samples were sheared at 150–450 rpm using spindle number 1. Rheological behavior was elucidated using plots of RPM versus viscosity.

#### 2.2.3. *In Vitro* Release

*In vitro* drug release studies on gelled formulations were carried out in triplicate using Franz diffusion cell. Parchment membrane (thickness 10 *μ*m) was placed between donor and receptor chamber of the cells with a contact area of 3.14 cm^2^and 2 g gel containing 10 mg of drug was placed in the donor chamber. Receptor phase containing phosphate buffered saline at pH 6.4 (PBS pH 6.4) warmed to 37°C was kept constantly stirred throughout the experiment with the help of a magnetic needle. At predetermined time points, 1 mL samples were withdrawn from the receptor phase and replaced with PBS pH 6.4. The samples withdrawn were filtered; drug release was quantified by UV spectrophotometry at 292 nm and expressed as cumulative percent released versus time for the 8 hr duration of the study. The release data beyond one hour (i.e., after the initial burst) was subjected to analysis for elucidating the mechanism of release.

#### 2.2.4. Mucoadhesion

The mucoadhesive force was determined using modified two-pan balance for both sol and gel states. The left hand side of the balance was provided with Teflon blocks at the top and the bottom and the right side had a receptacle for water. Mucin films were prepared on coverslips by placing 20 *μ*L of 3% w/v porcine mucin in simulated nasal secretion on a perfectly horizontal surface and air drying the films. During measurement, the films were hydrated for a minute with a drop of simulated nasal secretion (the ionic composition of simulated nasal secretion included 150 mM Na^+^, 41 mM K^+^, and 4 mM Ca^2+^ [18]). The coverslips were attached to the Teflon blocks with mucin containing sides facing each other using double sided adhesive foam tape. The test formulation was placed between the two coverslips balanced on the left pan of the balance. They were placed as sol at 25°C and were also gelled at 37°C prior to testing. Water was promptly added into the receptacle placed in the right pan at a rate of 5 mL/min using a peristaltic pump. Weight in grams of water required to separate the two surfaces was measured and mucoadhesive force was calculated as

(1) $$\begin{array}{c} F=\;W\times g, \end{array}$$

where *F* is the mucoadhesion force (dynes/cm^2^), *W* is the minimum weight required to break the mucoadhesive bond, and *g* is the acceleration due to gravity (cm/s^2^). The data was subjected to one-way ANOVA with Bonferroni's multiple comparison test at a significance level of *P* < 0.001.

#### 2.2.5. Assessment of Ciliary Function Using Frog Palate

Mucociliary transport time was determined* ex vivo* as an indicator of mucociliary function [19]. Movement of opium poppy seed along frog palate treated with formulations or controls served to measure mucociliary transport time. The protocol for assessment of ciliary activity in frogs was approved by Institutional Animal Ethical Committee (IAEC), No. 242, and the experimental procedure was performed in accordance with the CPCSEA guidelines. Frogs (*Rana breviceps*) were pithed by bending the head forward and inserting a needle into the brain and then down to the spinal cord. The jaw was disarticulated and the upper portion of the head was removed by cutting it with scissors from the junction of the posterior pharynx and esophagus out to the skin of the back. The palate was supported on a plastic board and introduced in a transparent chamber maintained at a relative humidity of 96% generated using saturated potassium chloride solution and the palate surface was observed through the chamber [20]. Control values were obtained for each experiment by applying 0.2 mL of control solution (PBS pH 6.4) to palate, leaving it in contact for 5 min, and then draining off. The gelled formulations were also applied onto the palate using spatula and were allowed to be in contact with the palate for 5 min followed by thorough rinsing of the palate with control solution. Immediately after the treatment, an opium poppy seed was placed over the middle portion of the palate using forceps and the time required for the seed to travel a 6 mm distance on palate was recorded in seconds. The effect of individual excipients on mucociliary clearance was also screened. The procedure was performed in replicate. The data was compared statistically using one-way ANOVA with Bonferroni's multiple comparison test at a significance level of *P* < 0.05.

#### 2.2.6. *Ex Vivo* Permeation Studies across Sheep Nasal Mucosa

For the purpose of this study, fresh nasal tissue was carefully removed from the nasal cavity of sheep at the local slaughterhouse with prior permission from concerned authorities at Deonar Abattoir, Mumbai. The nasal mucosa was separated from septum and the connective tissue as well as most of the adhering cartilaginous tissue was carefully removed with forceps and scissors without damaging or scratching the nasal mucosa. The separated mucosa was preserved in phosphate buffered saline pH 6.4 during transportation and was used within 4 hrs after the animals were slaughtered. The specimen were individually placed on Franz-type diffusion cells and clamped between the donor and receptor compartments. Seventeen milliliters of PBS pH 6.4 maintained at 37°C was used as the receptor phase. After a preincubation time of 15 minutes, 1 mL of either saturated doxepin solution in PBS pH 6.4 or C-GP-Dox and C-GP-PEG-Dox formulations equivalent to 10 mg of doxepin were placed in the donor compartment. Sampling was done in a similar manner as for* in vitro* release studies. Similarly drug free gels were also subjected to permeation studies to rule out any interference due to leaching from tissues over a period of time. The amount of Dox released into the receptor phase from the formulations was determined by UV at 292 nm. The method was found to be sensitive enough for detecting the amount of drug permeated and selective as adjudged from absence of significant absorbance from the release medium of blank gel subjected to release studies. The cumulative percent of Dox permeated versus time (hrs) graphs were plotted for both the gels and drug solution. The data was compared statistically using one-way ANOVA with Bonferroni's multiple comparison test (*P* < 0.001). Apparent permeability coefficient [Papp] for doxepin was calculated from the permeation data according to the following equation:

(2) $$\begin{array}{c} \text{Papp}=\frac{Q}{A*c*t}, \end{array}$$

where Papp is the apparent permeability coefficient (cm/s), *Q* is the total amount permeated throughout the incubation time (*μ*g), *A* is the diffusion area of the diffusion cell (cm^2^), *c* is the initial concentration of the drug in the donor compartment (*μ*g/cm^3^), and *t* is the total time of the experiment.

#### 2.2.7. *In Vivo *Activity of Doxepin

The protocol for animal testing was approved by Institutional Animal Ethical Committee (IAEC), No. 242. Animal care and handling throughout the experimental procedure were performed in accordance with the CPCSEA guidelines.* In vivo* efficacy of the formulations was evaluated in Swiss albino mice by means of measurement of the duration of immobility and activity counts in the animals using a forced swim apparatus. Mice (either sex) were randomly divided into four equal groups (*n* = 6). While the first group received isotonic saline and served as control, the three treatment groups received doxepin formulated as C-GP-Dox gels, C-GP-PEG-Dox gels, and as solution (prepared by dissolving 1.3 mg drug in 5 mL isotonic saline), respectively. Dose of formulations/solution to be administered to the mice was estimated as follows.

The reported effective intranasal human dose is 1 mg [21].

Considering average weight of humans and mice to be 70 kg and 20 g, respectively, the nasal dose for mice was calculated as per the conversion factor based on body surface area as

(3) $$\begin{array}{c} \text{Mouse}\;\text{dose}=\text{human}\;\text{dose}\times 0.0026=2.6\;\mu \text{g}. \end{array}$$

About 0.5 *μ*L of the formulations prepared in our studies would correspond to this dose. Since this volume was found to be too small for accurate measurement and administration to the mice a diluted version was prepared by simply dissolving only 0.26 mg Dox per mL of formulation without altering the composition of the gel. The test drug solution was also prepared in the same strength. A volume of 5 *μ*L per nostril of the formulations and drug solution were administered amounting to the calculated dose of 2.6 *μ*g of doxepin in mouse.

In all the animals, formulation equivalent to 2.6 *μ*g of drug was administered intranasally using micropipette. Administration of the drug was done by holding the animal in a supine position beneath the dosing probe of micropipette while a known droplet volume was placed above the external nares. This droplet was then allowed to be breathed into the nasal cavity while the animal was still in the supine position. The animal was repositioned, and the other naris was treated with the same droplet volume. The dosing procedure required was 10 to 15 seconds per animal.

Activity counts and immobility time were recorded by placing the animals in forced swim apparatus. The apparatus consists of a small water wheel set in a water tank. The animal is placed on this apparatus inside the wheel. The animal turns the wheel vigorously but when the animal abandons attempts to escape from the water the wheel stops turning. The number of rotations of the water wheel is counted by a digital counter. The immobility time is the time for which the animal remains in water without making any movements or attempts to escape and was counted manually using a stopwatch.

The mice were subjected to initial pretest swimming session by placing them in the apparatus for 15 min which induces a state of despair and this was followed by 5 min test period (without dosing). The activity counts and immobility time during 5 min test period were noted and this was considered as basal reading. The mice were then dosed once a day with intranasal formulations/solutions for 13 consecutive days. On 11th, 12th, and 13th day, immobility and the activity counts during 5 min test period were noted at time intervals of 30 min, 2 hr, and 5 hr after dosing. The data over the three-day test period (11th, 12th, and 13th day) was pooled and analyzed statistically by two-way ANOVA using Bonferroni's multiple comparison test and *P* < 0.001 was considered significant.

#### 2.2.8. Histopathological Study of Nasal Tissue in Mice

Nasal tissues of mice used for* in vivo* efficacy testing of formulations were histopathologically examined for damage/irritation due to the formulations. On termination of the efficacy studies, one Swiss albino mouse from each group was randomly selected for histopathological evaluation. The selected animals were further dosed once daily for two more consecutive days resulting in a total exposure of 15 days. The animals were sacrificed by cervical dislocation and decapitated. The head was fixed with 10% neutral buffer formalin for 48 hours. The bones were decalcified using 5% formic acid (Gooding and Stewart's solution) treatment for 10 days. After 10 days, nasal tissues were separated, washed with water, and processed for histopathological observation with different grades of alcohol (70%, 90%, and 100%), xylene, and paraffin. The tissues were embedded in paraffin blocks, mounted on a microtome, sectioned at a thickness of 4 microns on clean glass slides, and stained with haematoxylin-eosin. The slides of control and treated nasal mucosal tissues were examined using a light microscope for any lesions or damage to the nasal epithelium. Photographs of nasal mucosal tissues were also taken. The observations were scored depending on the extent of severity. The sections of nasal mucosa from either formulation treated or drug solution treated mice were compared with those from the control group.

## 3. Results and Discussion

### 3.1. Preparation of Gels

Chitosan is readily soluble in dilute acidic solutions below pH 6.0 and typically precipitates above this pH value. At low pH, the amino groups of chitosan are protonated resulting in aqueous solubility; but as pH of chitosan solutions is raised above 6, amino groups become deprotonated and the polymer loses its charge and becomes insoluble. However, careful addition of GP, a weak base to a cold acidic chitosan solution, neutralizes the positively charged ammonium groups on chitosan chains gradually. This leads to two phenomena.

Firstly, the precipitation of chitosan is prevented and polymer remains in solution even at higher pH values due to strong electrostatic attraction between ammonium groups of chitosan and phosphate groups of GP, thus maintaining solubility of chitosan even at near neutral pH. Secondly, the resultant mixtures show reversible gelation on warming. The glycerol groups of GP promote hydration of chitosan chains and, therefore, the polymer chains are stretched in solution at lower temperatures. On increasing the temperature, the change in entropy causes increased restructuring of free water by the glycerol moiety of *β*-GP, dehydrating chitosan chains. This causes increased interchain hydrophobic attraction over interchain electrostatic repulsion between chitosan chains and thermally induced transfer of protons from chitosan amine groups to the phosphate moiety of *β*-GP resulting in thickening of the sols and transformation to gels [22].

*Gelation Characteristics*

### 3.2. Gelation Time, Gelation Temperature, and pH

Both the ability of chitosan to remain in solution at higher pH and thermosensitivity require a critical GP content which varies with the degree of deacetylation and the molecular weight of chitosan (~73,000) [23]. Preliminary studies in our laboratory revealed that a minimum concentration of 8% of GP was essential for inducing thermoreversible properties to a 2% w/v chitosan solution. A 10% GP containing gel was found to show optimum gelation characteristics and pH. However, this concentration of GP results in a hypertonic solution. Since isotonicity is a vital property for nasally administered formulations, PEG 4000 was examined as an additive to facilitate preparation of thermally gelling chitosan solution at lower concentration of GP. Preliminary studies revealed the ability of PEG to preserve the gelling properties of the sol at reduced GP concentration. The sols containing PEG did not show gelation soon after preparation but gelled at physiological temperature when cured for 2 hrs by storage at 4°C. PEG has terminal hydroxyl groups which interact with the amino groups of chitosan in the presence of GP. GP may reduce the repulsion between similar charges in the chitosan chain and increases flexibility of chitosan chains, thereby allowing interaction between PEG and chitosan. This results in gelation even at lower concentration of GP. The interaction seems to hold true only over a very narrow GP concentration range (Table 1).

### 3.3. Rheological Evaluation

**Figure 1:**
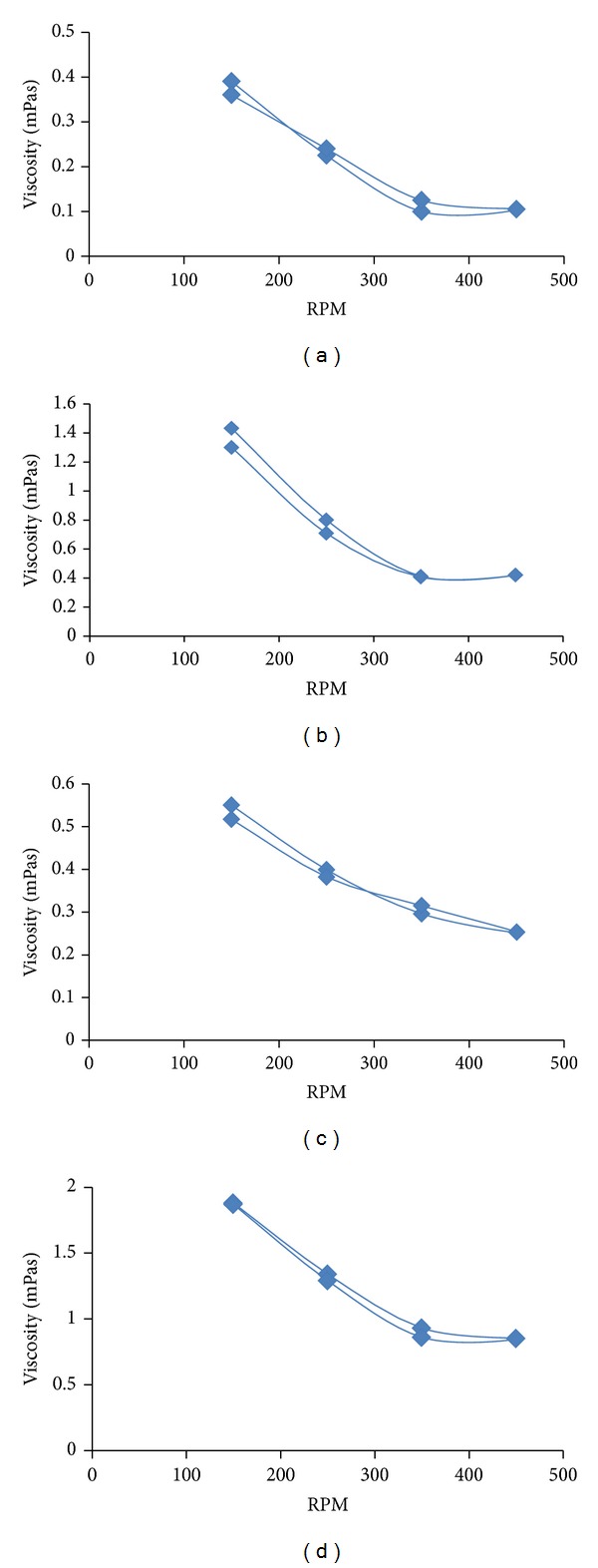
Rheological behavior of (a) C-GP-Dox and (b) C-GP-PEG-Dox gels at 25°C and (c) C-GP-Dox and (d) C-GP-PEG-Dox gels at 37°C.

From the rheological study it was observed that the formulations showed shear thinning behavior at both 25°C as well as at 37°C (Figures 1(a), 1(b), 1(c), and 1(d)). The systems also exhibited mild thixotropy. Also over the entire range of shear at which the measurements were carried out, the viscosity of C-GP-PEG gels was at all the measured points higher than C-GP gels at both temperatures.

### 3.4. In Vitro Release

**Figure 2:**
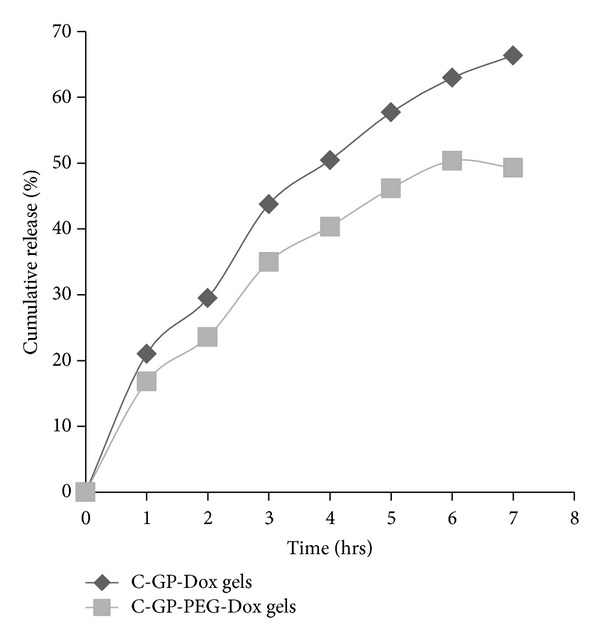
*In vitro* release of Dox from C-GP-Dox and C-GP-PEG-Dox gels.

During the release studies, the gels were found to remain intact on the surface of the parchment membrane at the end of the 8 hr study. An initial burst release of 20–25% of incorporated drug was observed from all formulations especially during the first one hour. The burst release could be attributed to drug that is not entrapped and distributed in the tunnel of the gel during gelation process which diffuses out rapidly. Following this, the drug entrapped into the hydrogel was released gradually from C-GP gels. About 70% of drug was released in 8 hrs. Addition of PEG decreased burst and slowed down the release of the drug from the gel and only about 50% Dox was released at the end of 8 hrs from C-GP-PEG gels (Figure 2). The C-GP-PEG gels have higher viscosity than C-GP gels at 37°C; therefore these gels form a matrix with a compact network and, hence, slower drug release was observed. Beyond the burst release, the drug release from both C-GP and C-GP-PEG formulations seemed to follow Higuchi's kinetics for diffusion controlled release since the correlation coefficient for this profile was the closest to unity.

### 3.5. Mucoadhesion

**Figure 3:**
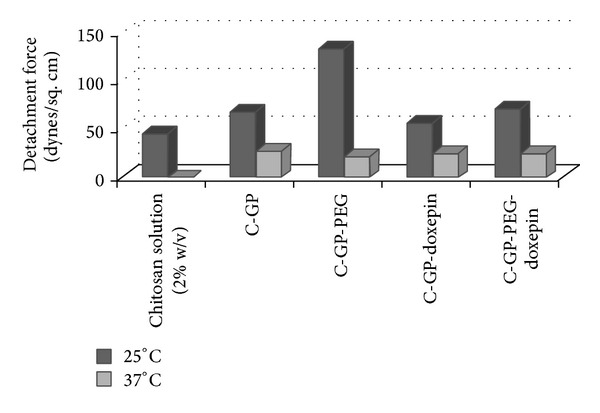
Comparison of mucoadhesive strength of different formulations.

Chitosan is a mucoadhesive polymer, the mucoadhesion being attributed to the electrostatic attraction between the positively charged D-glucosamine units of chitosan and the negatively charged sialic acid and sulphate residues of mucin [24]. At 25°C, all formulations showed mucoadhesion comparable to or greater than that of chitosan solution (Figure 3). In the sol state, the chitosan chains are freely available for interaction with the mucin resulting in good mucoadhesion. Addition of drug had no significant effect on mucoadhesion of C-GP formulations.

The PEG containing gels exhibited stronger mucoadhesive forces. This may be attributed to the fact that the formulations containing PEG have high chain flexibility and viscosity and interaction with mucin is higher and, therefore, at 25°C the highest mucoadhesive strength was observed for C-GP-PEG blank gels. However, in this case, loading of the drug resulted in a twofold decrease in mucoadhesion possibly due to an enhanced interaction between chitosan and PEG chains which are accompanied by increased viscosity of the gel as observed from the rheological measurements, which in turn reduce its binding to mucin.

Unfortunately, significantly lower mucoadhesive strength was recorded at 37°C for both formulation types. The formulations with and without drug failed to retain the adhesiveness on gelling. The mucoadhesive strength of both gels at 37°C was similar to no significant difference. During gelling, the polymer chains interact among themselves (through electrostatic forces) to form gel network rather than exhibiting interaction with mucin leading to the drop in mucoadhesive force measured.

### 3.6. Assessment of Ciliary Function Using Frog Palate

Mucociliary clearance depends on the quantity and viscoelastic properties of mucus, and number, ciliary beat frequency, and coordination of cilia [25]. Frog model has been shown to produce good correlation with* in vivo* tracheal clearance in mammals [26]. Chitosan is reported to have a transient inhibitory effect on mucociliary clearance [27]. Chitosan results in adhesive bond formation with mucosa and may impart increased resistance to beating of cilia thereby decreasing the clearance of the formulation. Also, chitosan in acidic solution is known to bind cations [28]. The free electronic doublet of nitrogen, which is present in the polymer, is responsible for the sorption of many cations, especially divalent cations. The high hydrophilicity of chitosan owing to the large number of hydroxyl groups and the flexible polymeric chain structure which favors the adjustment of the cations dispersed in solution for complex formation aid in the cation binding capacity of chitosan. Since calcium and magnesium play a role in regulation of ciliary activity (controls sliding of microtubules) [24], decrease in transport can also be attributed to sequestering of Ca^2+^ and Mg^2+^ ions and hence decreased ciliary beating.

The investigations showed that PBS (pH 6.4) and drug solution did not significantly affect ciliary beating. The transport rate of seed for palate treated with individual excipients and formulations was lower when compared with the time taken for the seed to move on untreated palate. The increased clearance times might be attributed to hypertonicity of the formulations and excipients. Hypertonic solutions are known to slow down or inhibit the movement of cilia.

**Figure 4:**
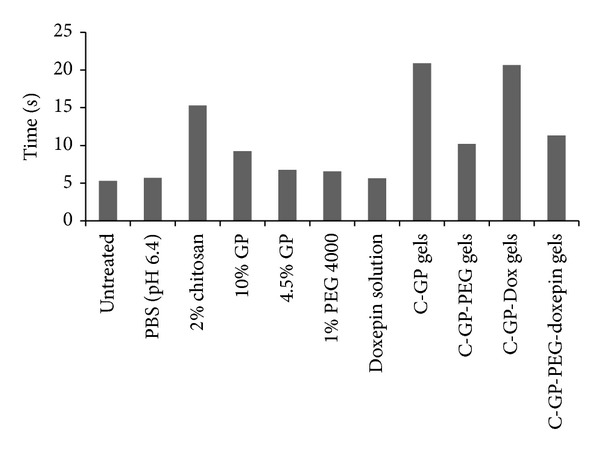
Effect of various excipients and formulations on ciliary function of frog palate (*n* = 6).

All the formulations showed statistically significant reduction in mucociliary transport time indicative of ciliary inhibition/damage as in Figure 4. It was found that the time taken for the seed to move on palate treated with the C-GP-PEG both for blank and drug containing gels was twice as compared to movement of seed on untreated palate which shows significant effect on ciliary movement. Seed movement was completely arrested on palate treated with C-GP gels with and without drug. Although no attempts were made to assess reversibility of ciliary inhibition in the present studies ability of a formulation to bring about reversible slowing or halt of mucus transport may be desirable to increase the contact time between the drug and nasal mucosa which in turn would increase nasal residence time and hence absorption. The high concentration of GP resulting in a hypertonic formulation could be one of the contributing factors.

### 3.7. *Ex Vivo* Permeation Studies across Sheep Nasal Mucosa

Doxepin showed good permeation across excised sheep nasal mucosa from saturated aqueous solution. Papp values of doxepin (1.329 × 10^-6 ^cm/s) indicate suitability of the chosen candidate for intranasal delivery. The release and permeation patterns from C-GP gels and C-GP-PEG gels show similar trends. The Papp values for doxepin from C-GP-PEG gels are significantly less in comparison with Papp values for drug solution and C-GP gels.

Chitosan is known to be a permeation enhancer for nasal formulations. Chitosan has been shown to increase the paracellular transport of polar drugs by transiently opening the tight junctions between the epithelial cells without producing immune response [26]. A surprisingly high rate of permeation of the drug was observed from gel matrix of C-GP. There was a significantly higher value of apparent permeability from C-GP formulation in comparison to the PEG containing gels and also drug solution of doxepin with a significance level of *P* < 0.01. The higher permeability from C-GP gels was especially striking since the drug solution contained a 5-fold higher amount of drug. This clearly seems to indicate an enhancement of permeation of drugs when applied in the form of C-GP gels.

**Figure 5:**
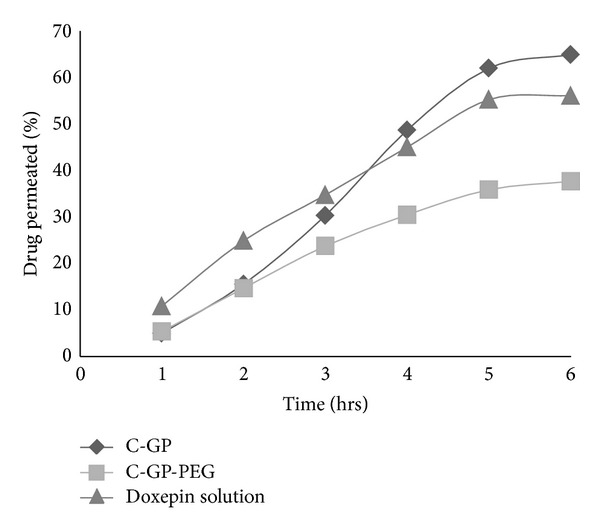
*Ex vivo* permeation across sheep nasal mucosa.

As evident from the figures, drug present in C-GP-PEG gel permeates at a rate lower than from the C-GP gels and drug solution as in Figure 5. The rate of permeation of doxepin from gel matrix would depend on the rate of its release from the matrix.

### 3.8. *In Vivo *Activity of Doxepin

The behavioral despair/forced swimming is based on the principle of measurement of the duration of immobility occurring following exposure of rodents to an inescapable situation. This behavior reflects a state of despair which can be reduced by several agents which are therapeutically effective in human depression. The majority of clinically used antidepressants decrease the duration of immobility [29].

Most antidepressants require a lag period of dosing ranging from 2 to 4 weeks for clinical effects of their administration to be apparent. Hence, in our present studies, the animals were dosed for 10 days before the actual measurements began in order to elicit a sufficient response of antidepressant activity [29].

In all cases, that is, mice treated with doxepin solution, C-GP-Dox, and C-GP-PEG-Dox, there was a significant increase in activity count and a decrease in immobility time indicative of good antidepressant activity.

**Figure 6:**
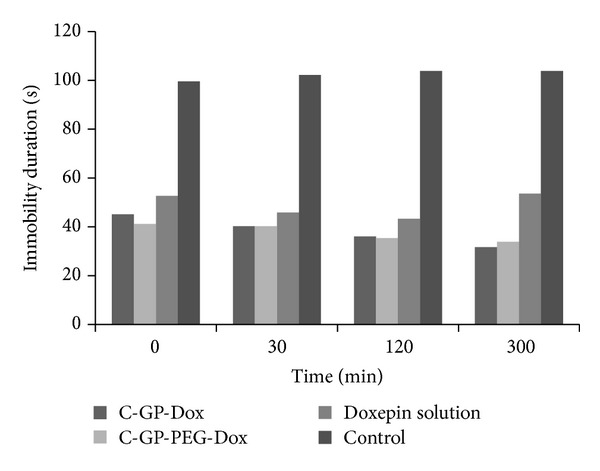
Immobility duration of Swiss albino mice after administration of formulations 13 days after dosing.

**Figure 7:**
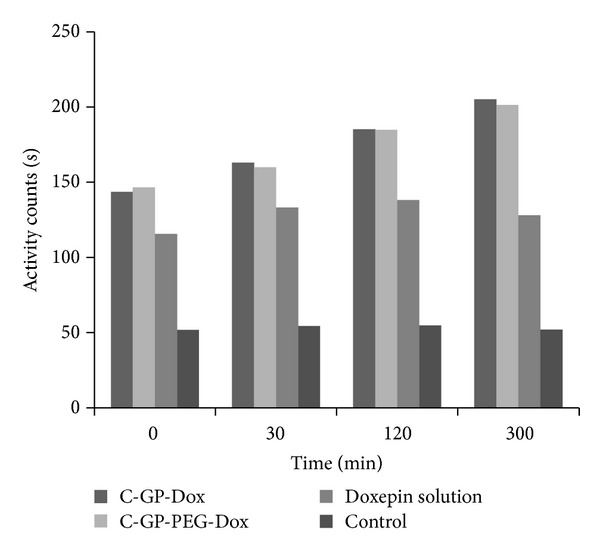
Average activity counts of Swiss albino mice on forced swim apparatus 13 days after dosing.

An unforeseen observation was the significant activity observed at time “0,” that is, promptly after administration of the gels; this could be attributed to the 10-day pretreatment of the animals with doxepin based formulations before the actual measurements were undertaken. Efficacy of the formulations was evident in the form of a gradual increase in activity counts and a decrease in immobility time over the 5-hour test period. The effect of formulations on activity counts and immobility time is shown in Figures 6 and 7.

The trend was similar for both formulations, and no apparent difference was distinguished between the two despite the* in vitro* differences observed in release rates.

Further, the measurements also revealed a difference between solution and formulation in terms of duration of action. Activity counts for the formulations were significantly higher and immobility time significantly lower than that from solution for the gel treated groups compared to solution at the end of five hours indicating sustained activity of gels.

### 3.9. Histopathological Study of Nasal Tissue in Mice

**Figure 8:**
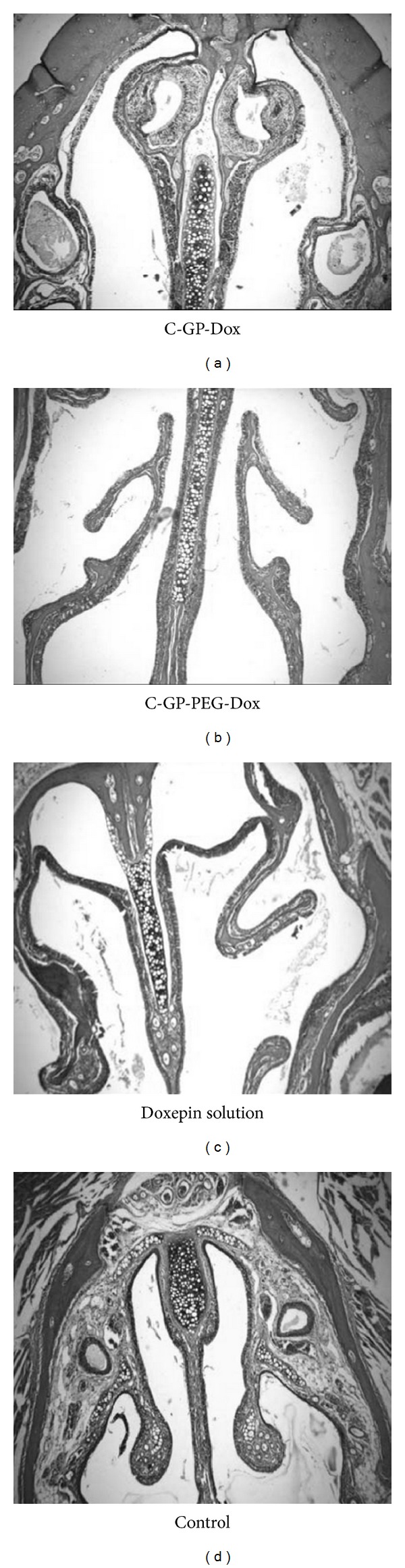
Histopathological study of nasal tissue in mice for group treated with doxepin containing formulations.

Nasal tissues of control group in which saline was administered showed no abnormality and there was no change in the nasal epithelium (Figure 8). Exposure of nasal tissues to drug solution apparently resulted in a significant damage to nasal mucosal tissues evidenced as moderate infiltration and glandular hyperplasia and severe epithelial hyperplasia. The sluffing caused by doxepin was reduced when drug was administered as gels. The treatment groups which received C-GP-Dox and C-GP-PEG-Dox showed only mild swelling of glands and inflammation (Table 2). Surprisingly, despite the hypertonicity associated with this system, the C-GP formulation seemed to be tolerated better with lowest damage to epithelial tissues. The results suggest good nasal acceptance of the drug in mice when formulated as chitosan based* in situ* gelling formulations.

## 4. Conclusion

The results presented here showed that chitosan/GP systems have the potential to be used as intranasal* in situ* gelling thermosensitive formulations for delivery of CNS acting agents since the systems possess optimum gelation characteristics, safety, and efficacy. In addition, due to the sustained effect of the formulations the dosing frequency can be reduced.

## Figures and Tables

**Table 1:** Gelation characteristics and pH of drug loaded C-GP and C-GP-PEG systems.

Formulation	Concentration of chitosan (% w/v)	Concentration of GP (% w/w)	Concentration of PEG (% w/w)	Gelation temperature (°C)	Gelation time (min)	pH
C-GP-Dox	2	10	—	37.4	7.32	6.93
C-GP-PEG-Dox	2	4.5	1	37	7	6.5

**Table 2:** Histopathological study of nasal tissue in mice.

Characteristic features	C-GP-DOX	C-GP-PEG-Dox	Dox solution
Glandular hyperplasia	Mild	Mild	Mild
Infiltration of the inflammatory cells	Mild	Mild	Mild
Epithelial hyperplasia	Mild	Moderate	Mild
Sluffing of mucosal epithelium	—	—	Mild
Congestion	—	—	—
